# Hyperspectral Imaging Analysis for the Classification of Soil Types and the Determination of Soil Total Nitrogen

**DOI:** 10.3390/s17102252

**Published:** 2017-09-30

**Authors:** Shengyao Jia, Hongyang Li, Yanjie Wang, Renyuan Tong, Qing Li

**Affiliations:** 1 College of Mechanical and Electrical Engineering, China Jiliang University, Hangzhou 310018, China; 15a0106134@cjlu.edu.cn (S.J.); 10b0102113@cjlu.edu.cn (H.L.); wangyanjiexx@163.com (Y.W.); tongrenyuan@126.com (R.T.); 2 College of Computer Science and Technology, Zhejiang University of Technology, Hangzhou 310014, China

**Keywords:** hyperspectral imaging, soil type classification, total nitrogen, texture features, data fusion

## Abstract

Soil is an important environment for crop growth. Quick and accurately access to soil nutrient content information is a prerequisite for scientific fertilization. In this work, hyperspectral imaging (HSI) technology was applied for the classification of soil types and the measurement of soil total nitrogen (TN) content. A total of 183 soil samples collected from Shangyu City (People’s Republic of China), were scanned by a near-infrared hyperspectral imaging system with a wavelength range of 874–1734 nm. The soil samples belonged to three major soil types typical of this area, including paddy soil, red soil and seashore saline soil. The successive projections algorithm (SPA) method was utilized to select effective wavelengths from the full spectrum. Pattern texture features (energy, contrast, homogeneity and entropy) were extracted from the gray-scale images at the effective wavelengths. The support vector machines (SVM) and partial least squares regression (PLSR) methods were used to establish classification and prediction models, respectively. The results showed that by using the combined data sets of effective wavelengths and texture features for modelling an optimal correct classification rate of 91.8%. could be achieved. The soil samples were first classified, then the local models were established for soil TN according to soil types, which achieved better prediction results than the general models. The overall results indicated that hyperspectral imaging technology could be used for soil type classification and soil TN determination, and data fusion combining spectral and image texture information showed advantages for the classification of soil types.

## 1. Introduction

Concerns about the environmental impacts of excessive nitrogen fertilizer application have been growing in recent years. In order to manage nitrogen in an efficient way and fertilize crops according to their demands, it is necessary to obtain detailed information about the total nitrogen (TN) of farmland soil. Traditional chemical analysis methods for TN are complex, time-consuming, costly and poor in real-time. A rapid, nondestructive method should be developed, which is a key step toward the successful implementation of precision farming. 

During the last two decades, near-infrared (NIR) spectroscopy has been widely employed as an effective tool for the analysis of soil properties. Numerous studies on the measurement of soil TN have been reported using this technique [1,2,3]. Nevertheless, the variability of the sample sets (soil texture, moisture content, minerals and organics) greatly complicates the prediction accuracy of the calibration models in soil near-infrared (NIR) spectroscopy analysis [4,5]. The spectral prediction mechanism may vary from one sample set to another. Due to this variability, the soil samples are first classified, then a local spectral model is established for each soil type which can effectively improve the prediction accuracy [6]. On the other hand, soil type classification is an important foundation of soil science, which provides the basis for rational exploitation and scientific management of soil resources. Many researchers have adopted NIR spectroscopy to distinguish soil types [7,8]. The correct classification can reach rates above 80% [9].

Developed from remote sensing, hyperspectral imaging (HSI) has gained extensive attention from different fields such as the food industry [10], agriculture [11], and medical science [12]. Through each measurement by the HSI instrument, both the spectral information and image texture information of the sample can be obtained. Image texture, which is characterized by the relationship of the intensities of neighboring pixels, has been successfully used for the classification of fruit ripeness [13], fish freshness [14], and plant disease degree [15], and the mapping of weed patches [16]. Cai, et al. used image texture features to classify soil samples with different degrees of salinization, and a higher correct classification rate was obtained [17]. They considered that when the soil samples were similar in spectral features, texture features would play a positive role in the sample recognition, and the combined spectral and texture features information can help to improve the classification accuracy. Ma, et al. used analysis of hyperspectral images to distinguish healthy, greening disease infected and zinc-deficient citrus [15]. As the leaf spectra of greening disease infected and zinc-deficient citrus were partially overlapped, and the leaf texture features of greening disease infected and zinc-deficient citrus were similar, the utilization of spectral information or texture features for modelling cannot achieve good classification results in this case, but data fusion combining spectral information and texture features greatly improved the correct classification rate for the three kinds of citrus. To our knowledge, comprehensive utilization of spectral information and image texture features for the classification of soil types has seldom been reported.

Hyperspectral imaging generates an immense amount of data. Some of them may contribute more co-linearity, redundancies, and noise than relevant information to calibration models, which is a huge challenge for the analysis of hyperspectral images [18]. Effective wavelength selection, aiming to select only a few wavelengths which carry most of the useful information with minimum collinearity and redundancy from full spectrum, is believed to reduce amount of data, computational task, and help build a simple and robust model [19,20]. The successive projections algorithm (SPA) is a popular tool for wavelength selection in multivariate calibration and classification [21]. It is able to select a small representative set of spectral wavelengths with a minimum of collinearity. He, et al. used a visible-near infrared HSI technique to detect the tenderness of Atlantic salmon, and SPA was applied to select effective wavelengths [22]. They stated that the number of wavelengths used in the calibration model can be significantly reduced without a decrease in prediction accuracy. In machine visual systems, the most popular method for texture feature analysis is gray level co-occurrence matrix (GLCM) method [23]. GLCM, created through calculating how often a pixel with a particular gray level value occurs at a specified distance and angle from its adjacent pixels, is able to take into account the specific position of a pixel relative to another. In this work, SPA and GLCM were adopted to select effective wavelengths and extract texture features, respectively. The objective of this work was to investigate the feasibility of classifying soil types and determining soil TN content using analysis of hyperspectral images. The specific objectives were to: (1) build classification models for soil types in utilization of spectral information and image texture features; (2) establish robust and accurate calibration models for each soil type to measure soil TN content.

## 2. Materials and Methods

### 2.1. Soil Samples and Laboratory Reference Measurement

The study area is located in the city of Shangyu (Zhejiang Province, People’s Republic of China, 29°43′38″–30°16′17″ N, 120°36′23″–121°6′9″ E), stretching 60 km from north to south and 40 km from east to west. The climate of this area is subtropical monsoon with an annual average temperature of 16.4 °C and mean annual precipitation of 1400 mm. The southern region of Shangyu is mainly hills and mountains. According to the classification and codes for Chinese soil (National Standard of China, GB/T 17296-2009), the most representative soil type of this region is red soil. The landforms of northern region are river network plains and coastal plains, and the main soil types are paddy soil and coastal saline soil, respectively. 

A total of 183 soil samples were sampled from different farmlands of 12 towns in Shangyu, including 84 paddy soil samples, 57 red soil samples and 42 seashore saline soil samples. They were taken from the upper soil layer (0–30 cm) from 2014 to 2016. The samples were collected using a soil-sampling auger. A composite sample was obtained by mixing five soil samples of equal volume, one from the central plot and the remaining four separated by 1 m from each other. To reduce the impact of soil moisture, the soil samples were tiled on a plate and air dried at 80 °C for 60 h. At the 48th h and the 60th h, three samples were randomly selected for weighing. Their weight had barely changed. Then the samples were sieved with a diameter of 1 mm. After that, the samples were air dried again at 60 °C for 48 h to reduce the impact of air moisture during storage. A small portion of each sample was sent to the agricultural testing center of Zhejiang Provincial Academy of Agricultural Sciences (ZPAAS) for soil chemical analyses. The remaining samples were used for HSI measurement. Laboratory reference measurement of soil TN were performed using the Kjeldahl method, as described in Hesse, [24]. Soil TN content was expressed in percentage of its weight to the total weight of dry soil. 

### 2.2. Hyperspectral Image Acquisition

**Figure 1:**
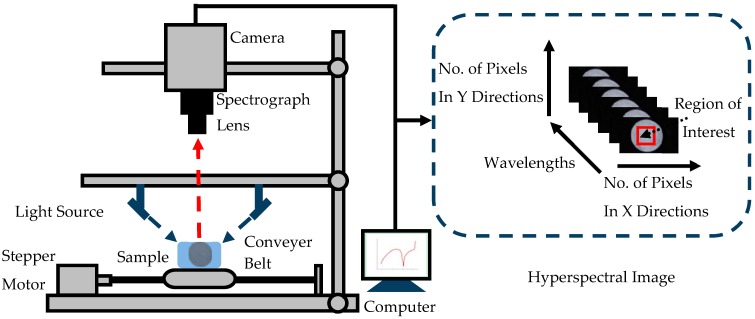
Schematic diagram of the hyperspectral imaging system. This system can obtain images in the spectral region of 874–1734 nm.

The hyperspectral images of soil samples were captured by a near-infrared HSI system with the wavelength range of 874–1734 nm and 256 bands. The system was composed of an imaging spectrograph (ImSpector N17E; Spectral Imaging Ltd., Oulu, Finland), a CCD camera (Xeva 992; Xenics Infrared Solutions, Leuven, Belgium), two 150W quartz tungsten halogen lamps (Fiber-Lite DC950 Illuminator, Dolan Jenner Industries Inc., Boxborough, MA, USA), and a conveyer belt which was driven by a stepper motor for sample movement (Figure 1). The entire system was fixed in a darkroom. The soil samples were put into Petri dishes with a diameter of 60 mm. The Petri dishes were placed on the conveyer belt for image acquisition. Hyperspectral image provided both spectral and image information simultaneously. Each pixel within the hyperspectral image contained a spectrum at the spectral range of the system, and there was a gray-scale image at each wavelength. 

To acquire clear and non-deformable hyperspectral images, the moving speed of the conveyer belt, the exposure time of the camera, and the height between the lens of the camera and the sample were set as 24 mm/s, 3 ms, and 30.8 cm, respectively.

The raw hyperspectral image (I0) was corrected by white (W) and dark (D) reference images. The white reference image was obtained using a standard Teflon tile (~99.9% reflectance), and the dark reference image was acquired by turning off the light source and covering the camera lens with its opaque cap. The corrected image (I) was calculated by the following equation:

(1) $$I=\frac{I_{0}-D}{W-D}\times 100\%$$

### 2.3. Spectral Data Extraction, Preprocessing and Effective Wavelength Selection

For each soil sample’s hyperspectral image, the region that covered the Petri dish without the edge was selected as the region of interest (ROI). The reflectance values of all pixels in the ROI were averaged to generate only one mean spectrum. Because of the noise in the head and the end of the spectra, only spectra at 975–1645 nm (200 bands) were used for further processing and model establishment. The same procedure was repeated for all ROI images, and a full spectrum matrix 183 samples × 200 bands was constructed. Standard normalized variate (SNV) was used to reduce baseline offset of the spectral matrix, and z-score normalization was used to get all the spectral data to approximately the same scale or to get a more even distribution of the variances and the average values [25].

Effective wavelengths were selected by the SPA method. Generally, SPA comprises two phases. The first phase consists of projections carried out on the spectral matrix, which generate candidate subsets of variables with minimum colinearity. In the second phase, candidate subsets of variables selected in the first phase are used to establish multi-linear regression (MLR) models. The best variable subset was determined on the basis of the root mean square error of leave-one-out cross validation in the calibration set (RMSECV). A detailed description of SPA can be found in literature [26,27].

### 2.4. Texture Variable Extraction

In creating the GLCM, the direction of 0°, 45°, 90° and 135° and distance of one pixel were applied, and four popular texture variables, such as energy, contrast, homogeneity and entropy were calculated in each direction based on GLCM [28,29]. The mean values of the four directions were used, and four averaged texture variables were obtained from the ROI of one gray-scale image. As the hyperspectral image contained gray-scale images at continuous wavelength bands, a total of 200 gray-scale images have been obtained from a single measurement of one soil sample. Extracting texture features from each gray-scale image would generate a large amount of redundant information which was not useful for modelling. Hence, texture features were only extracted from the gray-scale images at effective wavelengths. 

### 2.5. Establishment of Classification and Regression Models

**Figure 2:**
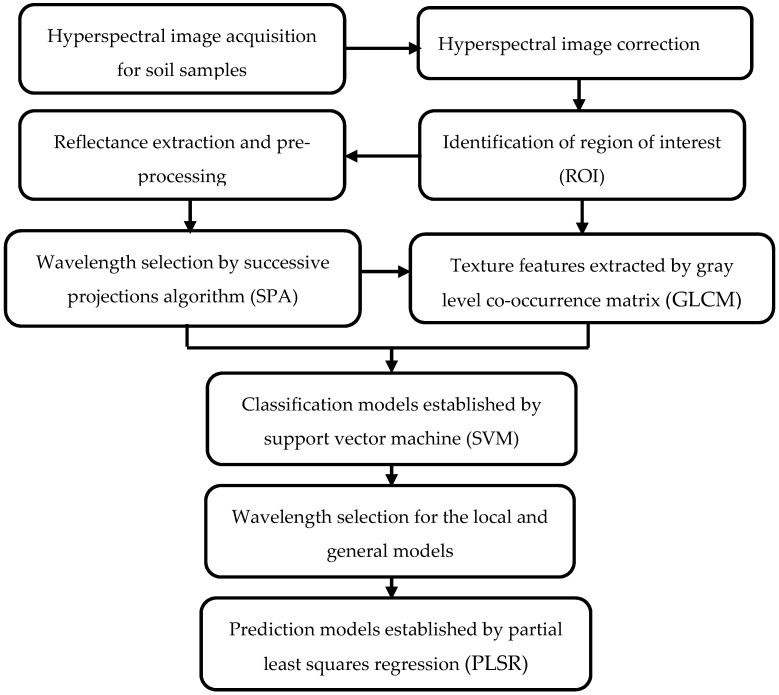
Main steps of this work.

The main steps of the work were shown in Figure 2. After hyperspectral image acquisition, correction and reflectance preprocessing, the samples of each soil type were randomly spilt into the calibration set and prediction set at a ratio of 2:1 so as to establish classification models: the calibration set was composed of 56 paddy soil samples, 38 red soil samples and 28 seashore saline soil samples, while the prediction set included the remaining 28 paddy soil samples, 19 red soil samples and 14 seashore saline soil samples. Then the SPA method was used to select effective wavelengths based on the calibration set. The reference data *y* in SPA was category value. The samples of paddy soil, red soil and seashore saline soil were assigned category values of 1, 2 and 3. After effective wavelength selection, texture features were extracted by GLCM. The method of support vector machines (SVM) was used to establish classification models based on the effective wavelengths and texture features. SVM has been proved as a reliable method for classification, dealing with both linear and nonlinear data efficiently [30,31]. In this work, radial basis function kernel was selected as the kernel function, which is the typical general-purpose kernel.

After soil type classification, both general and local models have been established for the prediction of soil TN content. In general models, the total samples were randomly divided into two subsets: the calibration set was composed of 122 samples, while the prediction set included 61 samples. In local models, the samples in each soil type were randomly divided into two subsets at a ratio of 2:1. The sample numbers in the calibration sets were 56, 38 and 28 for paddy soil, red soil and seashore saline soil respectively, while the prediction sets consisted of 28, 19 and 14 samples for paddy soil, red soil and seashore saline soil, respectively. The calibration sets was used to establish calibration models, whereas the prediction sets was used for independent prediction of the established models. The method of SPA was used to select effective wavelengths for each local model and the general models. In this procedure, the reference data *y* in SPA was soil TN content. The method of partial least squares regression (PLSR) was used to establish prediction models for soil TN based on full spectrum and effective wavelengths, which has been widely applied in many areas [32].

### 2.6. Performance Assessment and Software

The performance of the established models were evaluated by the root mean squared error of prediction in the prediction set (RMSEP), the residual predictive deviation (RPD) and the coefficient of determination (*R*^2^). Generally, large values of *R*^2^ and RPD, and small value of RMSEP indicate good performances. The hyperspectral image analysis was conducted on ENVI 4.6 (ITT, Visual Information Solutions, Boulder, CO, USA) and Matlab 2010 (The Math Works, Natick, MA, USA). The methods of SVM, SPA were operated in Matlab 2010, and the partial least squares regression (PLSR) models were established in Unscrambler 10.1 (CAMO Inc., Oslo, Norway).

## 3. Results and Discussion

### 3.1. Spectral Profiles

**Figure 3:**
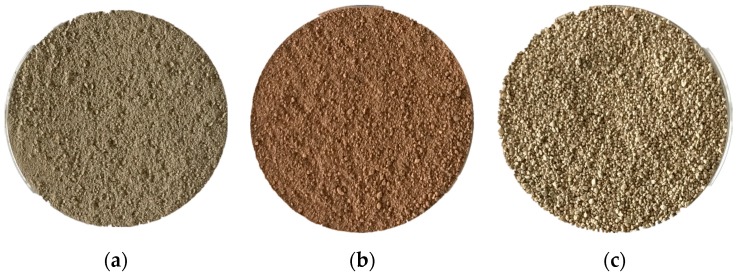
RGB images of paddy soil (**a**), red soil (**b**) and seashore saline soil (**c**) samples.

**Figure 4:**
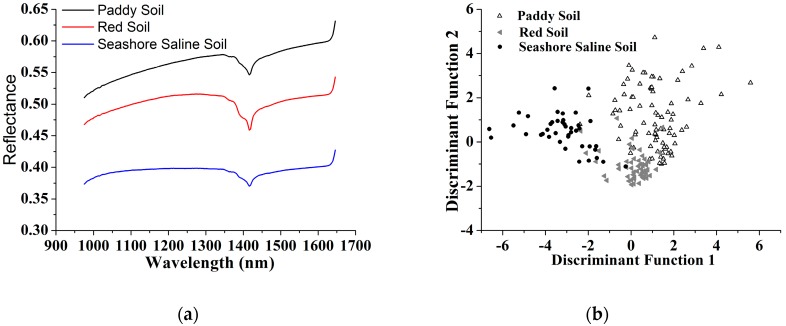
(**a**) The average spectrum of each soil type in the wavelength range of 975–1645 nm; (**b**) Grouping of 183 soil samples based on Fisher’s LDA using the first four principal components of full spectrum matrix as input.

Figure 3 shows RGB images of the three soil type samples. It can be noted that the surface of seashore saline soil was rougher than that of paddy soil and red soil. As can be seen in Figure 4a, the average spectrum of each soil type in the range of 975–1645 nm showed similar trend. Significant troughs appeared around 1400 nm in all spectra, which were attributed to the absorption of water in soil. There were some differences in the average spectral baselines. The reflectance value of seashore saline soil was lower than that of paddy soil and red soil, mainly because the light scattering of the surface of seashore saline soil was too intense. 

In order to examine the structure of the spectral data, a principal components analysis was performed on the full spectrum matrix. The principal components analysis scores were submitted to Fisher’s linear discriminant analysis (LDA). Because the first four principal components (PCs) of the spectral data can explain nearly 100% of total variance, they were set as input of LDA. Figure 4b shows the samples of paddy soil, red soil and seashore saline soil distinguished by the score plot of Fisher’s LDA. The correct classification percentage was 85%. It can be observed that the samples of paddy soil and seashore saline soil were relatively well grouped, while some red soil samples were mixed with the samples of the other two soil types.

### 3.2. Classification for Soil Types

**Figure 5:**
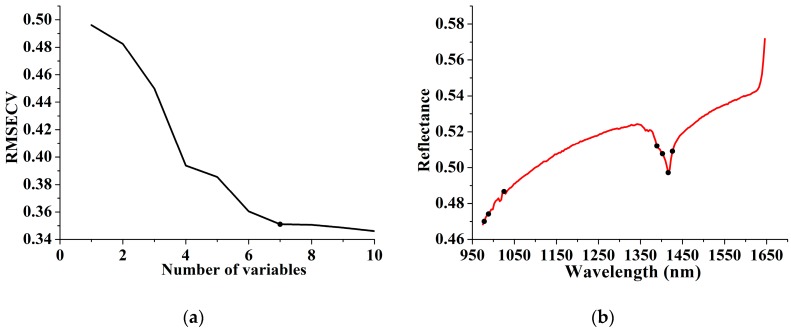
RMSECV curves with the number of variables selected by SPA for soil type classification (**a**). The reference data in SPA was category value. The selected variables (shown as dots) corresponding to raw spectra were presented in (**b**).

SPA was carried out to select effective variables from the full spectrum. The variation of RMSECV with the number of selected variables for soil type classification is shown in Figure 5a. Let RMSECVmin be the minimum value in the RMSECV sequence. Seven variables were selected through comparison of the RMSECV values which was not significantly larger than RMSEVmin by applying the F-test criterion with a significance level α = 0.25 [32]. Figure 5b presents an overview of the selected variables corresponding to raw spectra. The selected variables around the trough of 1400 nm can be approximately attributed to the absorption of water absorptions in the second overtone region, while the variables selected in the wavelength range of 950–1050 nm were related to overtones of aromatics C-H bond and amine N-H bond in organics [33]. This indicated that considerable differences existed in moisture content and organic ingredients among the samples of the three soil types.

ROI was defined as a rectangular area in the middle of the sample with 50 × 50 pixels (Figure 1). Four texture features (energy, contrast, homogeneity and entropy) based on GLCM at 7 effective wavelengths were extracted, resulting in a total of 28 texture features (4 texture features × 7 wavelengths) obtained from the ROIs for each soil sample. 

**Figure 6:**
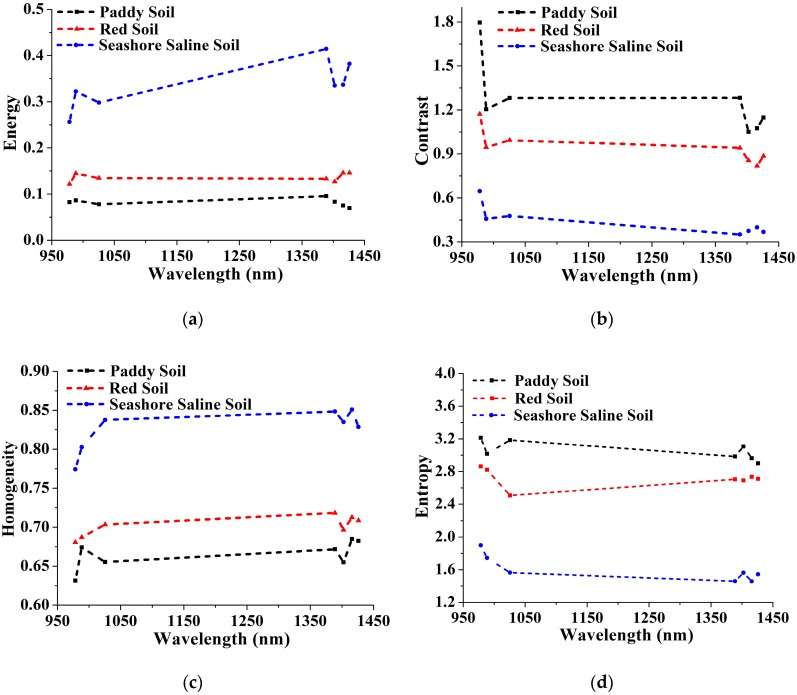
The mean values of (**a**) Energy, (**b**) Contrast, (**c**) Homogeneity and (**d**) Entropy of different soil types at the effective wavelengths.

Figure 6 shows the mean values of the four texture features of different soil types. It can be seen that energy and homogeneity of seashore saline soil was highest compared with the other two soil types at the effective wavelengths, which indicated that the image texture of seashore saline soil was rougher than that of the other two soil types [13]. A similar conclusion could be also obtained by analyzing the mean values of contrast and entropy. They were the lowest for seashore saline soil, which meant that the image texture of seashore saline soil contained less local variations. In general, the texture features of seashore saline soil were clearly distinguished from those of the other two soil types, and there were no intersections between the texture features of paddy soil and red soil, although they were close at some effective wavelengths. Hence, it was possible for soil type classification based on these statistics. 

To build SVM models for soil type classification, the samples of paddy soil, red soil and seashore saline soil were assigned category values of 1, 2 and 3. Table 1 showed the classification results of SVM models using different input variables. First, with full spectrum, the discrimination accuracy was 90.1% for the calibration set and 81.9% for the prediction set. When using spectral effective wavelengths for modelling, similar results were obtained for the calibration set and prediction set, respectively. It can be noted that the samples of paddy soil and seashore saline soil were well classified. Some of them were misclassified with red soil samples, while some red soil samples were misclassified with the samples of the other two soil types. The results were similar to those performed on the full spectrum matrix by LDA. Then, texture features were used for modelling. The discrimination accuracy was 81.9% for the calibration set and 77.0% for the prediction set. The performances were poorer compared with the model established by effective wavelengths. However, the samples of seashore saline soil were well classified from the samples of the other two soil types.

Finally, both effective wavelengths and texture features were set as input for building SVM models. As can be seen, the discrimination accuracy of the calibration set and prediction set were both improved compared with the models using only spectral effective wavelengths or texture features as input. The samples of paddy soil and seashore saline soil were successfully classified, while some samples of paddy soil and red soil were misclassified, and a few seashore saline soil samples were misclassified as red soil samples. The results indicated that data fusion by combining effective wavelengths and texture features showed advantages for the classification of soil types.

### 3.3. Prediction of Soil Total Nitrogen

The statistics values of soil TN content for the calibration sets and prediction sets in each local model and the general models were listed in Table 2. The concentration of soil TN ranged from 0.038% to 0.312%. The range of the prediction sets was covered in the calibration sets.

**Figure 7:**
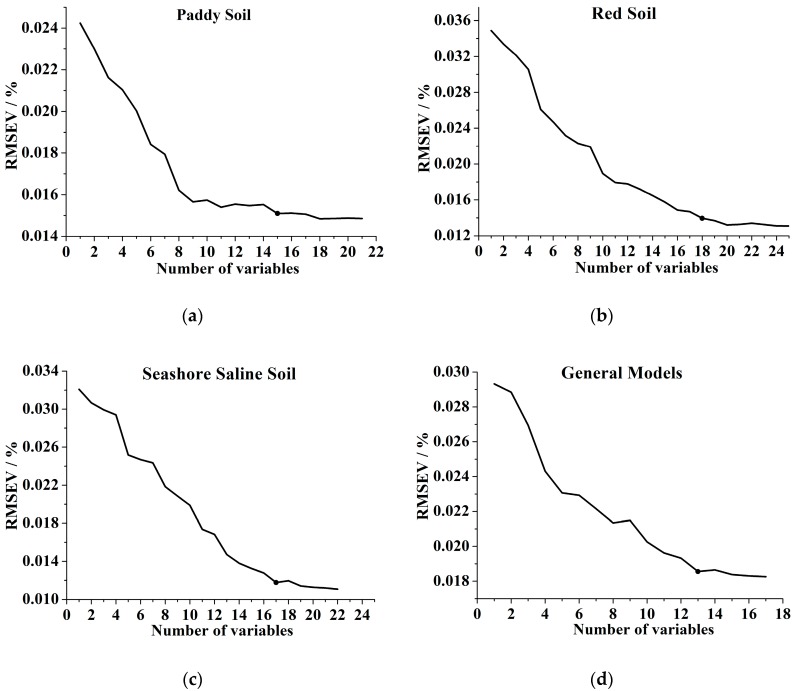
RMSECV curves with the number of variables selected by SPA for (**a**) paddy soil, (**b**) red soil, (**c**) seashore saline soil and (**d**) general models. The reference data in SPA was soil TN content.

The SPA method was used to select effective variables for the prediction of soil TN from the full spectrum. Figure 7 shows the variation of RMSEV with the number of selected variables for each local model and the general models. 

**Figure 8:**
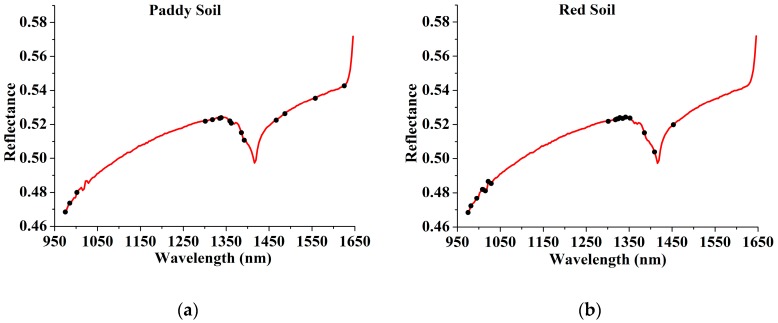

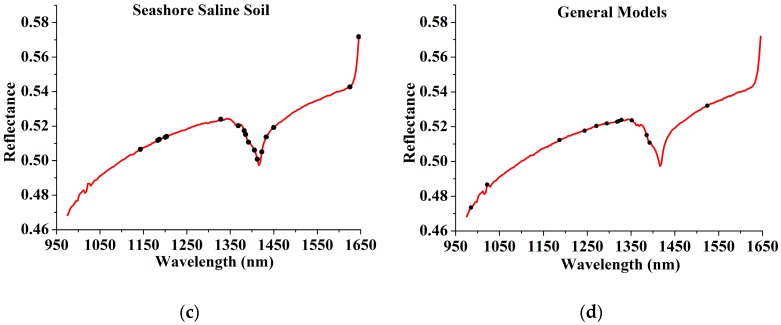
The variables selected by SPA (shown in circle markers) corresponding to raw spectra for paddy soil, red soil, seashore saline soil and general models were presented in (**a**–**d**), respectively.

Through comparison of the RMSEV values that were not significantly larger than RMSEVmin by applying the F test criterion with a significance level α = 0.25 [32], 15, 18, 17, and 13 variables were selected for paddy soil, red soil, seashore saline soil, and the general models, respectively. The overview of the selected variables corresponding to raw spectra is shown in Figure 8. The selected locations were mainly concentrated in the trough around 1400 nm and the wavelength range of 950 to 1050 nm. The result was supported by Yang et al. [2], who selected similar effective variables for soil TN in the NIR wavelength range. There were some differences among the selected locations for paddy soil, red soil, seashore saline soil and the general models, which indicated that the NIR feature absorptions varied from one soil type to another.

The prediction results of soil TN using different sample sets and input variables are listed in Table 3. Regression models were built by PLSR. It can be observed that whether using the full spectrum or effective wavelengths for modelling, the local models which were established individually for each soil type achieved better prediction results than the general models in terms of larger *R*^2^ and RPD values for the prediction sets. The reason can be attributed to the small variability of the samples in the same soil type [5]. Similar results have been reported by Mouazen, et al. [34], who developed local calibration models for the prediction of soil moisture content, so as to increase the accuracy of the NIR measurement. The full spectrum and effective-wavelength models achieved similar predictions for soil TN. However, the number of input variables in the effective-wavelength models was much reduced, and the efficiency has been promoted. 

## 4. Conclusions

In this work, a HSI system covering the spectral range of 874–1734 nm was used to classify soil types and evaluate soil TN content. The SPA method was applied to select effective wavelengths from the full spectrum, and texture features of energy, contrast, homogeneity and entropy were extracted from the gray-scale images at the effective wavelengths. The classification models for soil types and prediction models for soil TN were established by the methods of SVM and PLSR, respectively. The results showed that:

(1) The classification model established by the combining effective wavelengths and texture features data achieved the optimal results for the classification of red, paddy and seashore saline soil compared with the models established by the effective wavelengths or texture features alone. The correct classification rate was 91.8%.
(2) The soil samples were first classified, then local models were established for soil TN according to soil types, which achieved better prediction results than the general models.
(3) The overall results indicated that it was helpful to use image texture features for soil type classification, and HSI technique could be used for soil type classification and the determination of soil TN.

In future work, more soil samples with a wide range of soil types should be studied to build more robust soil type classification models and more reliable prediction models for soil TN. 

## Figures and Tables

**Table 1:** Classification results for soil types using SVM models established based on different input variables.

Input variables	(c, g) ^a^		Calibration Set					Prediction Set			
			1	2	3	Accuracy		1	2	3	Accuracy
Full spectrum	(30.94, 0.51)		52	3	1	92.8%		22	4	2	78.6%
			2	33	3	86.8%		2	15	2	78.9%
			1	2	25	89.2%		0	1	13	92.8%
		total				90.1%					81.9%
Effective wavelengths	(23.12, 2.42)	1	51	4	1	91.1%	1	24	2	2	85.7%
		2	3	33	2	86.8%	2	2	16	1	84.2%
		3	0	4	24	85.7%	3	1	2	11	78.6%
		total				88.5%					83.6%
Texture features	(90.95, 0.26)	1	47	9	0	83.9%	1	23	5	0	82.1%
		2	10	27	1	71.1%	2	7	12	0	63.1%
		3	1	1	26	92.8%	3	1	1	12	85.7%
		total				81.9%					77.0%
Effective wavelengths and texture features	(190.12, 2.28)	1	54	2	0	96.4%	1	27	1	0	96.4 %
		2	3	34	1	89.4%	2	3	16	0	84.2%
		3	0	1	27	96.4%	3	0	1	13	92.8%
		total				94.2%					91.8%

^a^ (c, g) are the parameters of the SVM model, where c is the penalty coefficient, and g is the kernel function parameter.

**Table 2:** Statistics of reference values of total nitrogen (TN) in the local models and general models.

Property	Calibration Set				Prediction Set			
	NS ^a^	Range (%)	Mean (%)	SD ^b^	NS	Range (%)	Mean (%)	SD
Paddy soil	56	0.088–0.312	0.170	0.036	28	0.124–0.255	0.174	0.042
Red soil	38	0.056–0.262	0.151	0.041	19	0.102–0.215	0.179	0.030
Seashore saline soil	28	0.038–0.205	0.131	0.031	14	0.055–0.178	0.133	0.030
General models	122	0.038–0.312	0.160	0.041	61	0.042–0.250	0.156	0.037

^a^ NS = Number of samples. ^b^ SD = Standard deviation.

**Table 3:** Comparison of prediction results for soil nitrogen using different sample sets and input variables.

Property	NS ^a^	Input Variables ^b^	NV ^c^	RMSEP ^d^	*R* ^2 e^	RPD ^f^
Paddy soil	28	Full spectrum	200	0.0166	0.83	2.5
		EW	12	0.0155	0.85	2.7
Red soil	19	Full spectrum	200	0.0129	0.80	2.3
		EW	10	0.0136	0.77	2.2
Seashore saline soil	14	Full spectrum	200	0.0118	0.83	2.5
		EW	10	0.0125	0.81	2.4
General models	61	Full spectrum	200	0.0176	0.76	2.1
		EW	14	0.0179	0.74	2.1

^a^ NS = Number of samples for the prediction set; ^b^ EW = effective wavelengths; ^c^ NV = Number of variables for the established models; ^d^ RMSEP = Root mean squared error of prediction in the prediction set; ^e^ *R*^2^ = Coefficient of determination; ^f^ RDP = Residual predictive deviation.
